# Ticks Associated with Macquarie Island Penguins Carry Arboviruses from Four Genera

**DOI:** 10.1371/journal.pone.0004375

**Published:** 2009-02-05

**Authors:** Lee Major, May La Linn, Robert W. Slade, Wayne A. Schroder, Alex D. Hyatt, Joy Gardner, Jeff Cowley, Andreas Suhrbier

**Affiliations:** 1 Queensland Institute of Medical Research, Brisbane, Queensland, Australia; 2 Southern Cross University, Lismore, New South Wales, Australia; 3 CSIRO Australian Animal Health Laboratory, Geelong, Victoria, Australia; 4 CSIRO Livestock Industries, Brisbane, Queensland, Australia; University of Liverpool, United Kingdom

## Abstract

Macquarie Island, a small subantarctic island, is home to rockhopper, royal and king penguins, which are often infested with the globally distributed seabird tick, *Ixodes uriae*. A flavivirus, an orbivirus, a phlebovirus, and a nairovirus were isolated from these ticks and partial sequences obtained. The flavivirus was nearly identical to Gadgets Gully virus, isolated some 30 year previously, illustrating the remarkable genetic stability of this virus. The nearest relative to the orbivirus (for which we propose the name Sandy Bay virus) was the Scottish Broadhaven virus, and provided only the second available sequences from the Great Island orbivirus serogroup. The phlebovirus (for which we propose the name Catch-me-cave virus) and the previously isolated Precarious Point virus were distinct but related, with both showing homology with the Finnish Uukuniemi virus. These penguin viruses provided the second and third available sequences for the Uukuniemi group of phleboviruses. The nairovirus (for which we propose the name Finch Creek virus) was shown to be related to the North American Tillamook virus, the Asian Hazara virus and Nairobi sheep disease virus. Macquarie Island penguins thus harbour arboviruses from at least four of the seven arbovirus-containing genera, with related viruses often found in the northern hemisphere.

## Introduction

**Figure 1:**
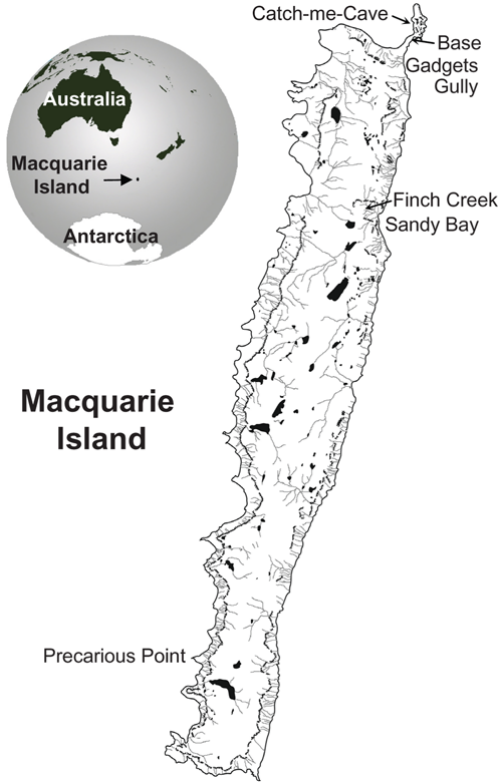
Map of Macquarie Island and its location on the globe. The locations of the Penguin colony sites where ticks were collected and the Antarctic Division base are shown.

Macquarie Island is a small (34 km long and 5 km wide) subantarctic island, which lies between Australia and Antarctica, 1,466 km SSE of Tasmania and 1,294 km N of the Antarctic continent (54°30′S, 158°55′E) (Fig. 1). The island is the only Southern Ocean biosphere reserve within the Man and the Biosphere Program of the United Nations Educational, Scientific and Cultural Organization (UNESCO). In 1997, Macquarie Island plus the surrounding 12 nautical miles of ocean became a world heritage area for geological and aesthetic reasons. The Australian Antarctic Division currently maintains a base on the island, which is resupplied twice yearly by sea. A number of tourist vessels also call for short visits each southern hemisphere summer. Macquarie Island is home to millions of seabirds, including four species of penguins; King (*Aptenodytes patagonicus*), rockhopper (*Eudyptes chrysocome*), royal (*Eudyptes schlegeli*) and gentoo (*Pygoscelis papua*) penguins. The only place in the world where royal penguins breed is Macquarie Island. The populations of these species have recovered well since Macquarie Island was declared a sanctuary in 1933 and the rendering down of penguins for oil ceased [1].

Arboviruses are distributed globally and belong mainly to the genera alphavirus (family *Togaviridae*), flavivirus (family *Flaviviridae*), nairovirus (family *Bunyaviridae*), phlebovirus (family *Bunyaviridae*), orbivirus (family *Reoviridae*), coltivirus (family *Reoviridae*) and vesiculovirus (family *Rhabdoviridae*). Each of these genera contains viruses, which are a significant cause of human and animal diseases globally. Arboviruses of the Antarctic region have not been studied extensively [2] and sequence information is available for only two, the flavivirus, Gadgets Gully [3], [4] and the Southern Elephant Seal alphavirus [5]. Given the rise in Antarctic tourism, the emergence and re-emergence of arboviral disease globally and the general concern for wildlife [6], [7], [8], [9], [10], we undertook to resurvey the tick-borne viruses associated with the different penguin species on Macquarie Island.

## Results

### Collection of ticks

*I. uriae* ticks were found under rocks near a King penguin colony at Sandy Bay, near a Royal penguin colony at upper Finch Creek, and inside Catch-me-cave (Fig. 1), a favoured roost of rockhopper penguins. Ticks could often be seen on King penguins, especially on young birds. Despite extensive searching, no ticks were found under rocks around the coastal gentoo penguin colonies near the Australian Antarctic Division base.

### Isolation of viruses and transmission electron microscopy

**Figure 2:**
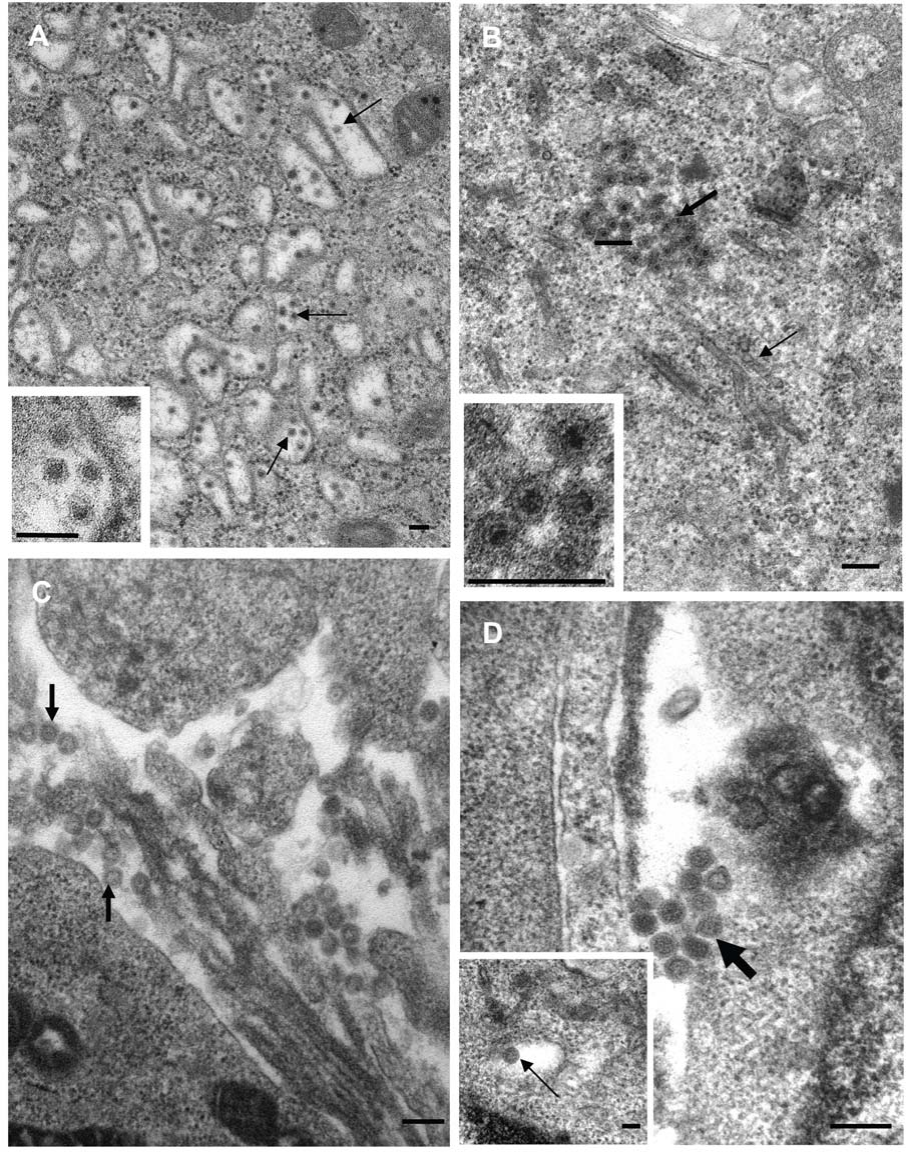
Identification of viruses by transmission electron microscopy. (A) F3/2 from a King penguin colony identified as a flavivirus after infection of BHK cells. Arrows indicate viral particles within cytoplasmic vesicles. Bars = 100 nm. (B) F3/4 from a King penguin colony identified as an orbi- or colti-virus (family *Reoviridae*) after infection of Vero cells. The thin arrow indicates a virus-specific tubule; the thick arrow aggregation of viruses, the associated electron semi-dense material represents material usually associated with viral assembly bodies. Bars  = 100 nm. (C) I2-19 from a rockhopper penguin colony identified as a bunyavirus after infection of Vero cells. Arrows indicate extracellular viruses. Bar = 200 nm. (D) EB6 from a royal penguin colony identified as a bunyavirus after infection of Vero cells. Arrows indicated extracellular viruses. Bar = 200 nm. Inset shows virus within an intracellular vesicle. Bar  = 100 nm.

A summary of all the viruses isolated by cell culture is shown in Table 1. Of the 90 ticks processed from the King penguin colony at Sandy Bay, 7 yielded virus. By electron microscopy the virus from 2 ticks (F3/2 and F4 BM) were identified as belonging to the genus *Flavivirus* (Fig. 2A). These were consistently about 45 nm in diameter, contained an inner electron dense core, were enveloped and were present within cytoplasmic vesicles (Fig. 2A). The viruses isolated from the other 5 ticks from the King penguin colony were identified as belonging to the genus *Orbivirus* or *Coltivirus* (Fig. 2B). The viruses were ≈65 nm in diameter, were non-enveloped, isometric in shape and cytoplasmic in location. Semi electron dense material was associated with the viruses and is characteristic of orbivirus inclusion bodies [11]. Cytoplasmic structures resembling virus-specific tubules [12] were also observed (Fig. 2B). Of the 36 ticks processed from the rockhopper colony at Catch-me-cave, 2 yielded virus and both were identified as belonging to the family *Bunyaviridae* (Fig. 2C). The enveloped viruses were spherical and 80–100 nm in diameter. These viruses were observed budding into smooth surfaced cytoplasmic vesicles (Fig. 2D insert) or associated with the external aspect of the plasma membrane (Fig. 2D). For ticks from the royal penguin colony at Finch Creek, only 1 out of the 43 yielded virus, and this was also identified as belonging to the family *Bunyaviridae.*

**Table 1:** Viruses isolated from ticks associated with penguin colonies.

Virus code	Location	*I. Uriae* stage sex	Penguins species	CPE day		EM ID	Sequence ID	Name
				BHK	Vero			
F3/2	Sandy Bay	Female	King	+++ d4	ND	Flavi	Flavi	Gadgets Gully
F4 BM	Sandy Bay	Female	King	+++ d4	− d5	Flavi	Flavi	Gadgets Gully
F3/4	Sandy Bay	Female	King	++++ d1	+++ d2	Orbi/Colti	Orbi	Sandy Bay^a^
F1/11	Sandy Bay	Adult female	King	ND	++++ d2	Orbi/Colti	ND	-
F1/40	Sandy Bay	Adult female	King	++ d2	-	Orbi/Colti	ND	-
F2/21	Sandy Bay	Adult male	King	++++ d2	ND	Orbi/Colti	ND	-
F2-22	Sandy Bay	Adult male	King	++++ d2	− d5	Orbi/Colti	ND	-
I2-19	Catch-me-cave	Adult male	Rockhopper	ND	++++ d6	Bunya	Phlebo	Catch-me-cave^a^
I2-7	Catch-me-cave	Adult male	Rockhopper	ND	++++ d6	Bunya	Phlebo	Catch-me-cave^a^
EB-6	Upper Finch Ck	ND	Royal	− d11	+/− d13	Bunya	Nairo	Finch Creek^a^

a Names proposed for the new virus isolates. CPE-cytopathic effect. ID-identification. Gadgets Gully virus infected cells did not react with the pan-flavi antibody 4G2 (data not shown). (CPE; + <20%, ++ 20–40%, +++ 40–60%, ++++ 60–80%).

After 2–4 weekly passages the flaviviruses and orbi/coltiviruses produced overt CPE in BHK cells within 2–4 days, and the rockhopper-associated bunyavirus produced overt CPE in Vero cells within 6 days. The royal penguin-associated bunyavirus showed only mild CPE after 12–14 days in Vero cells (Table 1).

### The flavivirus from ticks associated with King penguins

**Figure 3:**
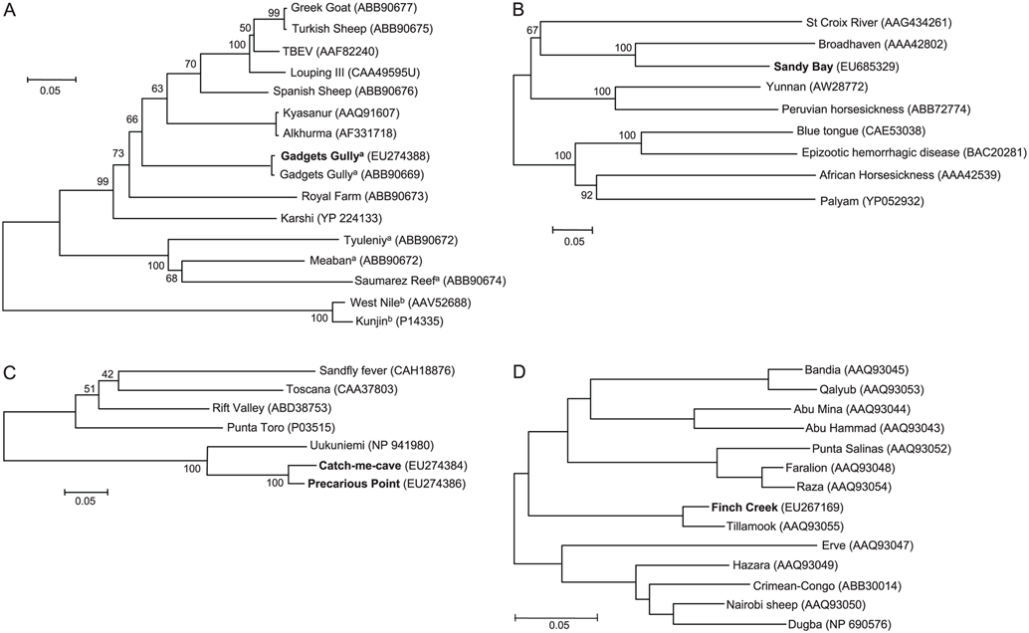
Dendograms showing the relationship between viruses. The scale shows the distance in terms of proportion of amino acids difference. The percent bootstrap support levels for each node are shown. (A) Gadgets Gully virus, genus *Flavivirus*. The dendogram was constructed from a 176 amino acid sequence of the E protein and recapitulates the dendogram shown previously for the complete polyprotein sequences [4]. Gadgets Gully in bold - virus collected in 2002. Gadgets Gully in normal text - virus collected in 1972. ^a^Seabird viruses. ^b^ Mosquito borne viruses. (B) Sandy Bay virus, genus *Orbivirus*. The dendogram was constructed from a 320 amino acid sequence of the VP5 outer capsid protein. Broadhaven, Nugget and possibly St Croix River virus [15] are tick born. (C) Precarious Point and Catch-me-cave virus, genus *Phlebovirus*. The dendogram was constructed from a 59 amino acid sequence of the N protein. Only a partial sequence was available for Precarious Point. Uukuniemi, Precarious Point and Catch-me-cave viruses are tick borne. (D) Finch Creek virus, genus *Nairovirus*. The dendogram was constructed from a 120 amino acid sequence from RNA polymerase and recapitulates the segregation previously reported between nairoviruses transmitted by soft ticks (Bandia to Raza) and hard ticks (Taggert to Dugba) [16].

The two flavivirus isolates from separate ticks collected at the Sandy Bay King Penguin colony (Table 1) were sequenced using primers based on Saumarez reef NS5 and E proteins [13]. The NS5 sequence (EU274387) showed a 96% sequence identity with nucleic acids 9307–9662 of the published Gadgets Gully NS5 gene (DQ235145.1), with no amino acid changes. The E protein sequence showed a 94% sequence identity with nucleic acids 1241–1659 of the published Gadgets Gully E gene (DQ235145) and resulted in only one conservative (I^454^ to V) substitution in the envelope protein (ABB90669). Phylogenetic analysis is shown in Fig. 3A and the closest relatives to Gadgets Gully virus are described in Table 2.

**Table 2:** Penguin virus genera and their closest relatives.

Virus name	Genus Family	Penguin species	Nearest relatives^a^	Known hosts	Location	Amino acid divergence %^b^
Gadgets Gully	*Flavivirus Flaviviridae*	King (Royal^c^)	Tick borne flaviviruses [4], excluding seabird viruses.	Mammals, humans	Northern hemisphere, Africa	Polyprotein^d^
						28–31%
						VP5
Sandy Bay^e^	*Orbivirus Reoviridae*	King	1. Broadhaven [42]	Sea birds	Scotland	40
			2. Yunnan [15]	Mice^f^	China	57.2
			3. Peruvian horse sickness	Horse	S. America	62.8
						N protein
Catch-me-cave	*Phlebovirus Bunyaviridae*	Rock-hopper	1. Precarious Point	Penguin	Macquarie	5.1
			2. Uukuniemi [24], [25]	Birds/mammals	Finland	32.7
			3. Rift valley fever	Ruminants	Africa/M. East	60.9
						N protein^g^
Precarious Point (Uukuniemi serogroup)	*Phlebovirus Bunyaviridae*	Royal^c^	1. Catch-me-cave	Penguin	Macquarie	5.1
			2. Uukuniemi	Birds/mammals	Finland	24.6
			3. Rift valley fever	Ruminants	Africa/M. East	57.4
						RNA pol
Finch Creek^e^	*Nairovirus Bunyaviridae*	Royal	1. Tillamook [16]	Unknown	N. America	4.6
			2. Hazara [43]	Rodents	Asia	23.1
			3. Nairobi sheep disease [44]	Goats/Sheep	Africa/Asia	24.6

a All viruses listed are tick borne except Rift valley fever virus. Underlined viruses or virus families are or contain, respectively, known pathogens of humans or animals.

b Based on amino acid identity.

c From ticks collected in 1972 near Royal penguin colony [3], [20].

d Based on complete polyprotein sequence [4].

e May be related to Nugget and Taggert virus [3], [20], respectively.

f Laboratory based experimental infection only [15].

g Based on partial sequence.

No significant neutralising activity was found in sera from 6 Antarctic division staff to our Gadgets Gully isolate or to Saumarez Reef virus (data not shown).

### The orbivirus from ticks associated with a King penguin colony

The EM results of the 5 viruses isolated from ticks associated with the King penguin colony at Sandy Bay suggested they were all orbi- or coltiviruses (Fig. 2). PCR primers based on conserved regions of VP3 from Broadhaven, Yunnan and St Croix River viruses failed to generate products (data not shown). Using the method of Potgieter *et al.* 2002 [14] to isolate and amplify viral dsRNA, several viral gene segments were cloned and sequence was obtained from the VP5 (EU685329), VP4 (Cap) (EU685332, EU685333), NS2 (ViP) (EU685331) and NS3 (EU685330) genes. BLAST searches illustrated that sequence for this or closely related viruses have not previously been reported; we thus propose the name Sandy Bay virus for this virus. Phylogenetic analysis using the VP5 protein sequence is shown in Fig. 3B and the nearest relatives are described in Table 2. NS3 sequence is also available for Broadhaven virus and showed a 85% amino acid sequence identity with Sandy Bay virus NS3 sequence (ACD38336), with Peruvian Horse sickness and Yunnan viruses again the next closest relatives (Supplemental Fig. S1). Sandy Bay virus also showed homology with Yunnan virus VP4 and NS2 (ViP) sequences (EU685333 and EU685331, respectively) with amino acid sequence identities of 40% and 32%, respectively (data not shown). Similar relationships to those shown in Fig. 3B were evident when VP4 sequences were used (data not shown). No sequence is available for Broadhaven virus VP4 and NS2 (ViP). The relationship between the close relatives of Sandy Bay virus and the rest of the viruses in the family *Reoviridae* have recently been described elsewhere [15].

### The phlebovirus from ticks associated with rockhopper penguins

Using our own (see Materials and methods) or published primers [16] for the nairovirus L polymerase gene, we were unable to obtain PCR products from the bunyavirus virus isolated from ticks associated with the rockhopper colony at Catch-me-cave. Using a primer, containing a 3′-terminus based on the conserved RNA segment termini of Uukuniemi virus, clones containing N (EU274386) and NS-s gene (EU274385) sequences of the S RNA segment of Precarious Point virus [3] were obtained. Using primers based on the Precarious Point virus sequence, N and NS-s gene sequences were obtained from the rockhopper penguin-associated virus (EU274384). The more conserved N protein sequence was used for phylogenetic analysis (Fig. 3C). The 5.1 % sequence divergence between the two viruses suggested the rockhopper penguin-associated virus was distinct from Precarious Point virus and we propose the name Catch-me-cave virus for this virus. The closest relatives for these viruses are described in Table 2.

### A nairovirus from ticks associated with the royal penguin colony

Sequence for the royal penguin-associated bunyavirus was obtained using newly designed degenerate PCR primers based on the conserved regions of nairovirus RNA polymerase genes [16]. The same or similar sequences have not previously been reported; we thus propose the name Finch Creek virus for this viral isolate. Phylogenetic analysis is shown in Fig. 3D and the closest relatives are described in Table 2.

## Discussion

The current study provides electron microscopic and sequence data for five arboviruses from four virus genera associated with Macquarie Island penguins and *I. uriae* ticks (summarized in Table 2). Together with the Southern Elephant Seal alphavirus [5], this island thus contains arboviruses from five of the seven genera of viruses known to contain arboviruses.

The nucleotide sequence similarity between Gadgets Gully virus isolated in the 1970's [3] and the isolate described herein suggest that this flavivirus has maintained remarkable genetic stability over ≈30 years, an observation consistent with the finding that flaviviruses have high fidelity RNA polymerases [17], [18]. Gadgets Gully virus has been referred to as a mammalian tick-borne flavivirus [4]. However, it has now been independently isolated twice from the seabird tick *I. uriae* associated with penguin colonies on Macquarie Island, suggesting that the enzootic host for Gadgets Gully virus is a seabird. Nevertheless, as shown previously [4], this virus does not group with the other seabird flaviviruses (Fig. 3A).

An orbivirus and a nairovirus have previously been isolated from *I. uriae* ticks associated with Royal Penguin colonies on Macquarie Island [19], [20]; Nugget virus (Kemerovo or Great Island virus serogroup) and Taggert virus (Sakhalin serogroup), respectively. As Broadhaven and Tillamook virus belong to the same serogroups [16], [21], [22], Sandy Bay and Finch Creek virus may be related to Nugget and Taggert virus, respectively. However, neither the viruses nor sequences from these latter viruses are available.

The observation that each virus was only associated with ticks from one penguin species might suggest that each virus is host specific. An alternative explanation is that fluctuating but nevertheless widespread immunity in these populations limits cycling of most of the viruses at any given time in any given bird population. As National Parks and Wildlife service has not allowed the trauma of blood collection from these recovering populations of nesting penguins, we were unable to resolve this issue. As arboviruses from these genera often have broad vertebrate host ranges [23], [24], [25], [26], [27], [28], and other seabird colonies have shown widespread immunity to multiple viruses from these genera [29], we would currently favour the latter explanation.

There is no currently indication that the penguin arboviruses pose a significant threat to the recovering penguin populations on Macquarie Island. To our knowledge there are currently only two reports of disease in penguins caused by tick-borne arboviruses from the genera described herein; (i) an uncharacterised arbovirus from *I. uriae* ticks from Macquarie Island (AUST-MI-411) was associated with disease and mortality in experimentally infected little blue penguins (*Eudyptula minor*) [9], and (ii) Humboldt penguins (*Spheniscus Humboldti*) and Black-footed penguins (*S. demersus*) have been fatally affected by West Nile virus [10]. Nevertheless, it is tempting to speculate that the gentoo penguins (unlike the other penguin species) move their nesting sites every year to avoid tick infestation [30] and perhaps also the associated arboviral infections. Interestingly, they are unique amongst Macquarie Island penguins in having no flavivirus antibodies [31].

The considerable sequence divergence of the viruses described herein from pathogenic viruses (Table 2) gives no indication that these penguin viruses pose an imminent threat to the health of humans or live stock. We and others [32] also failed to find evidence of human infection with Gadgets Gully or Saumerez reef virus. However, a serosurvey in the Australian Great Barrier Reef found that 4% of humans and birds were seropositive for Gadgets Gully [33]. Although no disease was reported [33], this study illustrated that Gadgets Gully virus can infect humans and has a range that includes tropical regions of Australia. In addition, tick borne arboviruses from seabirds have been reported to infect humans in Europe [34], and two seabird Sakhalin serogroup nairoviruses, Soldado and Avalon, have been reported to cause febrile illness and pruitus [35], and polyradiculoneuritis [36] in humans, respectively.

The rich arboviral diversity circulating in Macquarie Island penguins may be supported by the ideal transmission conditions associated with the crowded penguin nesting colonies. The geographic isolation of the island is probably not a barrier to the introduction of arboviruses as the extraordinary annual long distance migration of several seabird species may provide regular transcontinental transport of ticks and viruses. For instance, both sooty shearwaters [37] and Arctic Terns [38] visit Macquarie Island and migrate to all the continents of the northern hemisphere.

## Materials and Methods

### Tick collection

During the 6 day resupply of the Australian Antarctic Division base on Macquarie Island (12-16/3/02) by the RSV Aurora Australis, *I. uriae* ticks were collected from under rocks very close to three penguin colonies. An approach distance of no less than 5 m was maintained from the roosting birds at all times. Ticks were transported back to the base at ambient temperature, placed into Nunc vials, kept in a −70°C freezer at the base and on the return voyage, and were then couriered on dry ice from Tasmania to Queensland Institute of Medical Research, where they were stored at −70°C.

### Virus isolation

Individual ticks were placed into a plastic Petri dish containing 500 ul cold medium (RPMI 1640 supplemented with 5% FCS and 100 ug/ml streptomycin and 100 IU/ml penicillin) and were extensively chopped with two scalpel blades. The suspension was vigorously pipetted and then placed into an Eppendorf tube, which was spun at 8000 g for 10 mins at 4°C. Individual supernatants were added to BHK-21 (ATCC CCL-10) and/or Vero (ATCC CCL-8) cells at 1/10 and 1/100 dilutions. Cells were maintained as described [5]. The cells (10^5^ cells per 24 well) were cultured overnight before addition of tick extract, and the medium changed to RPMI 1640 supplemented with 2.5% FCS and antibiotics. When wells showed signs of cytopathic effects (CPE) ≈100 ul of supernatant was removed and placed onto fresh cells. After 3–5 passages viral stocks were prepared using cell grown in T75 flasks and were frozen in aliquots at −70°C.

Precarious Point virus (isolate CS0123) [3] was recovered from long term storage and had been passaged twice in suckling mouse brain and twice in BHK21 cells.

### Electron microscopy

Vero or BHK cells were infected with virus. When CPE was visible, the cells were fixed (1 h) in 2.5% (v/v) phosphate buffered (pH 7.2, 300 mOsmol/Kg) glutaraldehyde, rinsed in the same buffer (3×30 min) and then transferred to the CSIRO Australian Animal Health Laboratory where the cells were processed into ultrathin sections and examined by transmission electron microscopy as described previously [5].

### Virus sequencing

RNA was extracted from BHK or Vero cells (for the orbivirus) 2–3 days after infection (MOI≈1) using TRIzol reagent (Invitrogen). cDNA synthesis was performed using random hexamers, and Superscript III (Invitrogen) as per manufacturer's instructions. PCR amplification used 2 µl of cDNA, 200 nM primers and 2.5 U *Pfu* Turbo polymerase (Stratagene) as per manufacturer's instructions. Cycling conditions were 1 cycle of 95°C/1 min, 35 cycles of 95°C/30 sec, annealing at 40–55°C/30 sec and 68°C/5 mins. PCR products were gel purified using QIAquick Gel Extraction Kit (Qiagen). The purified PCR product was directly sequenced using Big Dye 3.1 sequencing mix (Applied Biosystems Inc).

The forward and reverse primers and annealing temperature used for the flaviviruses were 5′ GGATGGGGCAACCATTGTGG 3′, 5′ TCGTGCTGGCTTCCTGTTGG 3′ [13] and 50°C, for the E protein, and 5′ CCTACCACGCCAAGGTGGTCAG 3′, 5′ AGCARAAYGGGACCTCTTCCC 3′ and 55°C for NS5. The primer sequences for nairovirus L polymerase gene were forward 5′ GTIAGRAGYAARGTIRTITAIGA 3′ and reverse 5′ GCYTTIGGIGCYARIACIGCA 3′ or reverse 5′ GTRAARTCICCIGAIATRCA 3′ (annealing temperatures were 48 and 40°C, respectively). The primer sequences for the phlebovirus were based on sequence from Precarious Point virus and were forward 5′ GGCAGATGATGGACAGTGG 3′ and reverse 5′ GTCTGAGGAAGGCAAGAAGG 3′ (annealing temperature 55°C).

Precarious point virus RNA was amplified by RT-PCR using a single primer (5′-GCCGGAGCTCTGCAGAATTC*ACACAAAGAC*-3′) in which the 3′-terminal 10 nucleotides matched the conserved sequence at the RNA segment termini of Uukuniemi virus. cDNA was synthesised using 0.5 µg total RNA, primer and Superscript II reverse transcriptase (Invitrogen) and amplified by PCR using the same primer, *Taq* DNA polymerase (Promega) and 2.5 mM MgCl_2_. PCR conditions were 95°C/2 min, 10 cycles of 95°C/30 sec, 25°C/30 sec, 72°C/3 min, 25 cycles of 95°C/30 sec, 60°C/30 sec, 72°C/3 min and final extension at 72°C/10 min. DNA products were gel purified and cloned into pGEM-T vector and DNA from colonies containing inserts sequenced.

Sequence from the orbivirus was obtained by preparing dsRNA from infected cells as described [14]. Purified viral dsRNA (≈500 ng) was ligated to the PC3 primer [14] using T4 RNA ligase 1 (New England Biolabs). The primer tagged dsRNA was purified using QIAprep Spin Miniprep Kit (QIAGEN) and reverse transcribed using the PC2 primer [14] and Superscript(III) (Invitrogen). The complementary cDNA strands were annealed by heating to 95°C and cooling slowly to 72°C. PCR amplification was undertaken using *Pfu* Turbo polymerase (Stratagene) and the PC2 primer . PCR conditions were an initial step of 72°C/5 mins and 94°C/2 mins, followed by 40 cycles 94°C/15 sec, 58°C/30 sec and 72°C/2 min, and finally 72°C/5 min. PCR products were gel purified, cut with BamH1 and cloned into pEGFP-N1 using T4 DNA polymerase (Roche). Viral fragments were sequenced using pEGFP-N1 primers 5′ CAGAGCTGGTTTAGTGAACCGTC 3′ and 5′ CCGTCCAGCTCGACCAG 3′.

### Sequence analysis

Predicted amino acid sequences were determined using the Translate Tool ExPASy (http:au.expasay.org). The amino acid sequences were then used to query the non-redundant protein sequence database at NCBI using the blastp program (http://blast.ncbi.nlm.nih.gov/Blast.cgi). Alignments of related sequences were created using the ClustalW program [39] incorporated into the MEGA3 program [40], and then adjusted manually if necessary (the alignments are available on request). Distances between sequences were proportion of amino acid differences (using complete deletion). Phylogenetic reconstructions were done using the neighbour-joining method [41] as implemented in MEGA3. Bootstrap support levels at nodes were calculated after 500 replications.

## Supporting Information

Figure S1 (0.40 MB EPS)
